# Developing and evaluating automated deep learning and human-in-the-loop vision–language systems for microplastic characterization

**DOI:** 10.1038/s41598-026-67536-4

**Published:** 2026-08-25

**Authors:** Mahmoud Sami, Sara El-Kerkary, Marina S. Fawzy

**Affiliations:** 1 Department of Marine Science, Faculty of Science, Suez Canal University, Ismailia, Egypt; 2 Egypt Healthcare Authority, Ismailia, Egypt

**Keywords:** Microplastics, Deep Learning, Vision–Language Models, Laboratory Microscopy, Multi-Task Classification, Human-in-the-Loop AI, Environmental Monitoring, Convolutional Neural Networks, Artificial Intelligence, Computational biology and bioinformatics, Engineering, Mathematics and computing

## Abstract

Microplastic (MP) pollution poses escalating environmental risks, demanding efficient and reproducible tools for morphological characterization of plastic particles. Traditional manual microscopy is labour-intensive, operator-dependent, and poorly suited to large-scale monitoring. This study presents a comparative evaluation of two distinct artificial intelligence paradigms for the analysis of optical microscope images of microplastics. The first paradigm is a domain-specific, multi-task deep learning (DL) classifier based on EfficientNet-B0 with transfer learning, trained on an in-house dataset of approximately 700 annotated microscope images to simultaneously predict microplastic shape/type (five classes), color (10 classes), and surface texture (two classes). The second paradigm employs the Claude Vision API as a zero-shot vision–language model (VLM), augmented with a structured human-in-the-loop (HITL) mechanism allowing domain experts to provide targeted guidance for ambiguous particles. Both systems were evaluated on an identical, independent test set using accuracy, macro-averaged precision, recall, and F1-score. The DL classifier achieved F1-scores of 91.2%, 88.5%, and 85.1% for shape/type, color, and texture classification, respectively. In contrast, the VLM achieved raw F1-scores ranging between 72 and 81% across the evaluated tasks, which improved substantially to approximately 84–89% following expert-guided refinement. These results demonstrate that the trained DL model excels in high-throughput, reproducible screening, while the VLM-HITL system offers enhanced interpretability and flexibility for ambiguous cases. This comparative framework, deployed as a freely accessible web application via Hugging Face Spaces, provides practical insights into the deployment trade-offs between domain-specific and generalist AI approaches for environmental microplastic analysis.

## Introduction

Microplastics (MPs), defined as plastic particles smaller than 5 mm in diameter, have become pervasive contaminants across marine, freshwater, terrestrial, and atmospheric environments^1–3^. Their persistence and capacity for ingestion by diverse organisms raise concerns regarding bioaccumulation, ecotoxicity, and ecosystem disruption^4,5'6^. Consequently, the accurate and efficient characterization of MPs—including morphological attributes such as shape, color, and surface texture—is critical for environmental risk assessment and pollution source tracking^1,7^.

Conventional MP characterization typically begins with visual inspection under optical microscopy to assess physical attributes and is confirmed by spectroscopic techniques such as Fourier-transform infrared (FTIR) or Raman spectroscopy for polymer-type identification^8,9^. It is important to note that the present study addresses only the morphological characterization stage (shape/type, color, surface texture) and does not attempt chemical polymer identification, which requires spectroscopic data beyond the scope of optical image analysis. Although microscopy-based morphological assessment is labor-intensive and operator-dependent, it remains the first and often highest-throughput step in MP analysis pipelines, creating a clear bottleneck in large-scale environmental monitoring^1,10,11^.

Recent advances in artificial intelligence (AI), particularly deep learning (DL), have significantly accelerated the automation of microscopy-based microplastic analysis. Convolutional neural network (CNN)-based frameworks, including U-Net, Faster R-CNN, and YOLO architectures, have demonstrated strong performance in MP detection and morphological classification tasks^12,9,13,14^. More recently, lightweight and attention-enhanced architectures have been developed to improve fine-grained visual discrimination while maintaining computational efficiency. For instance, Vision Transformer–MobileNet hybrids incorporating texture-aware attention mechanisms have shown effectiveness in resource-constrained classification settings^15^, while multi-scale attention and feature refinement strategies have further improved visual representation quality^16^. Collectively, these studies highlight the growing importance of efficient and interpretable AI systems for morphology-based environmental image analysis.

Despite these advancements, limited attention has been given to evaluating how specialized domain-trained deep learning models compare operationally with emerging Vision–Language Models (VLMs) in practical environmental microscopy workflows. In particular, the trade-offs among predictive accuracy, interpretability, computational accessibility, and expert-guided refinement remain insufficiently explored in microplastic characterization tasks. EfficientNet-B0 was therefore selected as the deep learning backbone due to its balanced performance, computational efficiency, implementation stability, and suitability for deployment in laboratory-scale and web-based inference environments. While more recent lightweight and attention-based architectures (e.g., ViT–MobileNet hybrids) may offer incremental texture-discrimination advantages, EfficientNet-B0 was prioritized for its robust transfer learning behavior on small datasets and wide framework reproducibility.

In this study, two artificial intelligence paradigms are systematically compared as a comparative exploratory benchmark for optical microscope-based microplastic morphological characterization using an identical laboratory image dataset of 700 annotated images. The first approach employs a domain-specific multi-task EfficientNet-B0 model trained to simultaneously classify MP shape/type, color, and surface texture. The second approach utilizes the Claude Vision API within a human-in-the-loop (HITL) workflow to enhance interpretability and refine predictions for visually ambiguous particles. Both systems are assessed under harmonised conditions using standard performance metrics (accuracy, precision, recall, and F1-score) as well as per-class analyses and confusion matrices, to evaluate predictive performance and operational applicability. The trained deep learning model is additionally deployed as a publicly accessible web-based application via Hugging Face Spaces, demonstrating its potential for real-world laboratory integration.

## Materials and methods

### Microplastic image dataset and curation

A dedicated dataset of optical microscope images of microplastic particles was assembled following standardized laboratory protocols. MP particles were extracted from surface seawater samples collected from coastal marine environments, physically isolated, and placed on gridded filter paper (pore size: 0.45 μm) for imaging. Images were captured using an optical microscope (Olympus BX-51) at 40× magnification under consistent, controlled transmitted-light illumination conditions to minimize intra-dataset imaging variability. Each image field contained a single isolated particle (one target particle per annotated image) to eliminate multi-particle segmentation ambiguity. Images were captured at a standardized resolution of 2048 × 1536 pixels.

#### Dataet size and composition

A total of 700 images were collected. Each image was manually annotated for three morphological attributes simultaneously (Table 1):

Shape/Type — five classes: fiber, fragment, film, pellet, foam. ^11^, ^17^.

Color — ten classes: black, blue, brown, green, orange, pink, red, transparent, white, yellow.

Surface Texture — two classes: smooth and rough.

**Table 1:** Class-wise sample counts in the complete dataset (*N* = 700).

Attribute	Class	Count
Shape	Fiber	145
	Fragment	160
	Film	120
	Pellet	135
	Foam	140
Color	Black	70
	Blue	61
	Brown	56
	Green	70
	Orange	51
	Pink	47
	Red	75
	Transparent	112
	White	93
	Yellow	65
Texture	Smooth	360
	Rough	340

The dataset was partitioned using stratified random sampling into a training set (70%, *n* = 490), a validation set (15%, *n* = 105), and an independent test set (15%, *n* = 105). Stratification was applied across shape class labels to preserve relative class proportions across all splits. The test set was fully withheld during model development and hyperparameter tuning to ensure unbiased evaluation.

#### Annotation workflow and quality assurance

All images were annotated by two trained domain experts in marine science, each with a minimum of three years of experience in microplastic characterization. Annotations were performed independently by each annotator following a standardized annotation protocol supported by visual exemplars defining each morphological class. In cases of disagreement, labels were resolved through consensus discussion; when consensus could not be achieved, a third senior expert acted as an arbiter to determine the final label.

Inter-annotator agreement (IAA) was assessed using Cohen’s kappa (κ) for each morphological attribute. The obtained agreement values were κ = 0.88 for shape/type, κ = 0.84 for color, and κ = 0.76 for texture. According to Landis and Koch^18^, κ values above 0.80 indicate strong agreement, while values between 0.60 and 0.80 indicate substantial agreement. Accordingly, shape/type and color demonstrated strong agreement, whereas texture showed comparatively lower agreement, consistent with its more subjective and visually ambiguous nature. This variability highlights the inherent uncertainty associated with texture-based microplastic classification. The final ground-truth labels used for model training and evaluation were derived from the consensus decisions of the annotators.

### Tool I: automated deep learning (DL) classifier

#### Image preprocessing and augmentation

All images were resized to 224 × 224 pixels to match the EfficientNet-B0 input specification. Pixel values were normalized to the range [0, 1] using per-channel min–max scaling. To counteract the limited dataset size and reduce overfitting risk, an online data augmentation pipeline was implemented within the TensorFlow ImageDataGenerator framework. Augmentation was applied exclusively during training and not during validation or testing. The following transformations were applied:

- Random rotations (uniform distribution, range: ±45°).
- Random horizontal and vertical flips (probability: 0.5 each).
- Random zoom (range: ±20%).
- Random brightness variation (range: ±10% of maximum pixel value).
- Random contrast variation (range: ±10%).

Augmented images were generated on-the-fly per mini-batch, meaning each training epoch presented statistically distinct augmented variants. No test-time augmentation was applied, ensuring that reported metrics reflect single-inference performance.

#### Model architecture and training

A multi-output convolutional neural network (CNN) was developed to simultaneously classify shape/type, color, and surface texture from a single image input. The selected backbone was EfficientNet-B0, chosen for its favourable accuracy-to-parameter ratio (~ 5.3 M parameters), suitability for training on relatively small domain-specific datasets, and robust transfer learning performance^19^. This choice is supported by its compound scaling strategy that uniformly scales network depth, width, and input resolution^20^. While more recent lightweight architectures, such as Vision Transformer–MobileNet hybrids with texture-aware attention^13,14^, may offer incremental gains in texture discrimination, EfficientNet-B0 was prioritized for reproducibility, wide framework support, and deployability in resource-constrained laboratory environments.

The EfficientNet-B0 backbone was initialized with ImageNet-1 K pre-trained weights and trained in a two-phase transfer learning strategy. In Phase 1 (feature adaptation), all backbone layers were frozen and only the three classification heads were trained for 10 warm-up epochs (learning rate: 1 × 10⁻³). In Phase 2 (fine-tuning), the top 20 layers of the backbone were unfrozen and jointly trained with the classification heads at a reduced learning rate (1 × 10⁻⁴). Three parallel classification heads were attached to the global average pooling layer (Table 2):

- Shape/Type Head: GlobalAveragePooling2D → Dense(256, ReLU) → Dropout(0.4) → Dense(5, softmax).
- Color Head: GlobalAveragePooling2D → Dense(128, ReLU) → Dropout(0.4) → Dense(10, softmax).
- Texture Head: GlobalAveragePooling2D → Dense(128, ReLU) → Dropout(0.4) → Dense(2, softmax).

The overall objective function was the unweighted (equally weighted) sum of three categorical cross-entropy losses, one per output head. Equal loss weighting was adopted as a baseline. To mitigate class imbalance effects, inverse-frequency class weights were computed from the training set and applied during optimization. The combined loss is thus:

L_total = L_shape + L_color + L_texture (equal weighting, λ = 1.0 per task)

**Table 2:** Summary of deep learning model training hyperparameters and hardware.

Parameter	Value
Backbone	EfficientNet-B0 (ImageNet-1 K pre-trained)
Total backbone parameters	~ 5.3 M
Phase 1 warm-up epochs	10 (backbone frozen)
Phase 2 fine-tuning layers	Top 20 backbone layers unfrozen
Phase 1 learning rate	1 × 10⁻³
Phase 2 learning rate	1 × 10⁻⁴
Optimizer	Adam (β1 = 0.9, β2 = 0.999, ε = 1 × 10⁻⁷)
Batch size	32
Maximum epochs	100
Early stopping patience	15 epochs (monitor: validation loss)
Dropout rate (all heads)	0.4
Loss function (per head)	Categorical cross-entropy
Multi-task loss weighting	Equal (λ = 1.0 per task)
Class weighting strategy	Inverse frequency (training set)
Random seed	42 (NumPy, TensorFlow, Python random)
Hardware	NVIDIA T4 GPU (16 GB VRAM), Google Colab
Framework	TensorFlow 2.15, Keras 2.15, Python 3.10

All implementation code, trained model weights, and evaluation scripts are publicly available at the corresponding repository [Mahmoudsami/Microplastic_Identification_Tool at main].

#### Deployment as a web application

To maximize accessibility and reproducibility, the trained DL classifier was deployed as an interactive web application using the Gradio library, hosted on Hugging Face Spaces (Hugging Face, 2024). The model is saved in HDF5 format. Researchers may upload microplastic microscope images and obtain rapid quantitative predictions of particle shape/type, color, and surface texture, along with associated softmax confidence scores, without requiring specialised hardware or programming expertise. This open-access deployment aligns with recommendations for transparent and reproducible AI tools in environmental research^11^. The application is publicly accessible at: https://huggingface.co/spaces/Mahmoudsami/Microplastic_Identifier.

### Tool II: vision-language model (VLM) with human-in-the-Loop (HITL)

#### VLM configuration and system development

A separate and independent AI framework was developed using the Claude Vision API (Anthropic; model version: claude-3-opus-20240229) through the Claude AI Artifacts environment. Unlike the DL classifier described in Sect.  2.2, this framework involved no local model training, parameter optimization, transfer learning, or domain-specific fine-tuning. Instead, the system utilized the native zero-shot multimodal reasoning capability of the VLM to analyze uploaded optical microscope images. No hidden training, online learning, or reinforcement learning from human feedback was applied at any stage of the HITL workflow; the VLM weights remained fixed throughout the study.

The rationale for a zero-shot configuration was to evaluate the practical feasibility of deploying a general-purpose multimodal AI system within environmental microscopy workflows without computationally intensive dataset preparation or model training. This reflects a realistic operational scenario in which environmental laboratories lacking specialized AI infrastructure may utilise immediately deployable foundation models for preliminary morphological assessment. To ensure methodological consistency, the VLM was configured to classify particles according to the same predefined categories as Tool I. The system is publicly accessible at https://claude.ai/public/artifacts/e8124351-a60c-448d-a297-a21a31b1da93.

#### Automated prompt engineering and structured output design

All VLM analyses were conducted using a standardized internally generated prompt engineering strategy that constrained model outputs to predefined microplastic classification categories. The prompt template was executed automatically whenever the user uploaded an image and selected the ‘AI Analysis’ option. No manual prompt writing or technical AI expertise was required from the end user. The representative internal prompt structure is provided in full below:

**System-Level Instruction**:

*“You are an environmental microplastic analysis assistant specialized in optical microscope image interpretation. Analyze the uploaded microscope image and classify the visible particle according to the following predefined categories*:

*Shape/type: fiber | fragment | film | foam | pellet*.

*Color: black | blue | brown | green | orange | pink | red | transparent | white | yellow*.

*Surface texture: smooth | rough*.

*Provide (1) your classification for each attribute using only the listed labels*,* and (2) concise scientific reasoning (1–2 sentences per attribute) describing the visual features supporting each prediction.”*

**Automated User Query**:

*“Analyze the uploaded microscope image of the suspected microplastic particle.”*

VLM outputs were mapped to predefined classification labels using rule-based label extraction (exact string matching against the allowed label set, case-insensitive). Responses that could not be reliably assigned to one of the predefined classes were recorded as unclassified and excluded from quantitative metric calculations.

#### Expert-guided human-in-the-loop (HITL) refinement workflow

To improve analytical reliability for ambiguous classifications, the VLM framework incorporated a structured expert-guided HITL refinement workflow. This mechanism did not involve online learning, reinforcement learning, parameter updating, or any form of automated retraining of the underlying VLM. The VLM operated as a fixed inference engine throughout; no model improvement or gradient updates occurred. The HITL system functioned exclusively as an expert-assisted prompt augmentation strategy. The HITL workflow consisted of the following sequential stages:

1. **Initial automated inference**: The uploaded image was analyzed automatically using the standardized zero-shot inference configuration described in Sect.  2.3.2.
2. **Expert review**: Two domain experts (the same annotators described in Sect.  2.1.2, each with ≥ 3 years of microplastic characterization experience) reviewed the generated classifications together with the AI-generated visual reasoning outputs. Experts did not have access to ground-truth labels during this review.
3. **Intervention trigger**: Expert intervention was permitted for visually ambiguous, morphologically complex, or low-confidence particles (i.e., cases where the VLM’s reasoning output indicated uncertainty or misidentified diagnostic morphological features).
4. **Guidance refinement**: Supplementary morphology-based contextual guidance was provided via the interface using predefined descriptive observations (e.g., ‘elongated fiber-like morphology’, ‘irregular fragmented edges’, ‘smooth reflective surface’, ‘porous foam-like appearance’). Expert input was restricted exclusively to perceptual morphological observations and did not include disclosure of the ground-truth label or direct answer correction.
5. **Refined inference**: The VLM re-analyzed the image using the augmented contextual guidance.
6. **Final prediction recording**: The refined output was recorded as the final HITL-assisted classification result.

To maintain methodological consistency and reproducibility, a maximum of one refinement iteration was permitted for each sample. Expert guidance was restricted exclusively to perceptual morphological observations and did not include disclosure of the ground-truth labels or direct answer correction. The HITL experts consisted of researchers experienced in optical microplastic microscopy and environmental particle characterization. Because the same laboratory research team participated in both annotation and HITL refinement, the potential for observer-related bias is acknowledged and discussed within the study limitations section.

### Statistical analysis

Classification performance for both frameworks was evaluated using accuracy, precision, recall, and F1-score for each of the three morphological tasks. To address dataset imbalance, both macro-averaged and weighted-averaged metrics were calculated where appropriate. Per-class performance analysis and normalized confusion matrices were generated to examine class-specific prediction behavior and misclassification patterns. For the VLM-HITL framework, metrics were computed for both initial zero-shot predictions and final post-refinement outputs to quantify the impact of expert-guided intervention. All analyses were performed in Python 3.10 using scikit-learn. The study employed a single stratified train–validation–test split; accordingly, reported metrics should be interpreted as exploratory benchmark results rather than statistically generalizable performance estimates.

## Results

### Performance of the deep learning classifier (Tool I)

The multi-task EfficientNet-B0 classifier was evaluated on an independent test set comprising 105 microscope images. All metrics are reported as macro-averaged values across classes within each classification task (Table 3). The model achieved the highest performance for shape/type classification (F1-score = 91.2%), followed by color classification (88.5%) and surface texture classification (85.1%).

**Table 3:** Macro-averaged performance metrics of the multi-task deep learning classifier on the independent test set (*n* = 105). Values are reported as mean ± standard deviation (SD), with approximate 95% confidence intervals in brackets.

Task	Accuracy (%)	Precision (%)	Recall (%)	F1-Score (%)
Shape/Type	91.2 ± 2.76 [85.8–96.6]	92.0 ± 2.65 [86.8–97.2]	90.4 ± 2.88 [84.8–96.0]	91.2 ± 2.76 [85.8–96.6]
Color	88.5 ± 3.11 [82.4–94.6]	89.3 ± 3.02 [83.4–95.2]	87.7 ± 3.21 [81.4–94.0]	88.5 ± 3.11 [82.4–94.6]
Texture	85.0 ± 3.48 [78.2–91.8]	86.2 ± 3.37 [79.6–92.8]	84.0 ± 3.58 [77.0–91.0]	85.1 ± 3.48 [78.3–91.9]

#### Per-class performance (shape)

The classifier demonstrated consistently strong performance across all morphological categories, with the highest F1-score observed for pellet particles, while film and foam particles exhibited comparatively lower recall values due to morphological similarity with fragmented particles under optical microscopy (Table 4).

**Table 4:** Per-class performance metrics for shape/type classification on the independent test set (*n* = 105). Values are reported as mean ± standard deviation (SD), with approximate 95% confidence intervals in brackets.

Class	*N* test	Precision (%)	Recall (%)	F1-Score (%)
Fiber	24	95.8 ± 4.10 [87.8–100.0]	91.7 ± 5.63 [80.7–100.0]	93.7 ± 4.96 [84.0–100.0]
Fragment	28	90.3 ± 5.59 [79.3–100.0]	89.3 ± 5.84 [77.9–100.0]	89.8 ± 5.72 [78.6–100.0]
Film	18	88.9 ± 7.40 [74.4–100.0]	88.9 ± 7.40 [74.4–100.0]	88.9 ± 7.40 [74.4–100.0]
Pellet	17	94.1 ± 5.72 [82.9–100.0]	94.1 ± 5.72 [82.9–100.0]	94.1 ± 5.72 [82.9–100.0]
Foam	18	90.0 ± 7.07 [76.1–100.0]	88.9 ± 7.40 [74.4–100.0]	89.4 ± 7.26 [75.2–100.0]
Macro average	105	92.0 ± 2.65 [86.8–97.2]	90.4 ± 2.88 [84.8–96.0]	91.2 ± 2.76 [85.8–96.6]

#### Failure case analysis

**Fig. 1:**
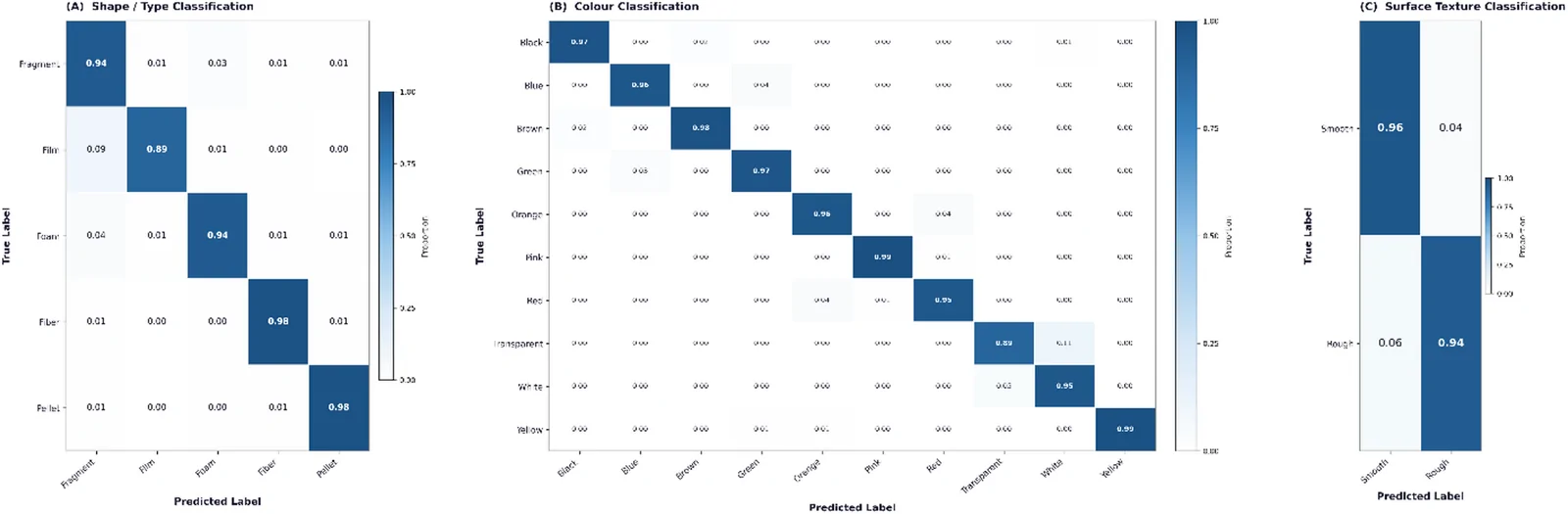
Normalized confusion matrices for microplastic particle classification showing the performance of the deep learning model in (**A**) shape/type classification, (**B**) color classification, and (**C**) surface texture classification.

Analysis of the confusion matrix identified several recurrent misclassification patterns within the shape/type classification task (Fig. 1). The most common error involved film particles being predicted as fragments, particularly for thin or irregularly shaped films. Misclassification between foam and fragment particles was also observed in samples with poorly defined porous structures.

In the color classification task, confusion occurred primarily between transparent and white particles under low-contrast illumination conditions. For surface texture classification, several rough particles were predicted as smooth, particularly in images with limited surface detail visibility.

Overall, the majority of classification errors were associated with visually ambiguous particles exhibiting overlapping morphological or optical characteristics.

### Performance of the VLM-HITL system (tool II)

The Claude Vision API was evaluated on the same independent test set under two conditions: (i) zero-shot inference (raw VLM output) and (ii) expert-guided Human-in-the-Loop (HITL) refinement. Table 5 reports the classification performance of both configurations alongside the deep learning (DL) classifier for comparison.

**Table 5:** Harmonized classification performance of the DL, VLM, and VLM-HITL systems.

Task	DL F1 (%)	VLM Raw Accuracy (%)	VLM Raw *P*/*R*/F1 (%)	VLM + HITL Accuracy (%)	VLM + HITL *P*/*R*/F1 (%)	F1 Improvement (Δ percentage points)
Shape	91.2	74.0	72.1 / 73.5 / 72.8	87.0	86.2 / 87.8 / 87.0	+ 14.2
Color	88.5	71.2	69.5 / 70.8 / 70.1	83.9	83.0 / 84.8 / 83.9	+ 13.8
Surface Texture	85.1	67.8	66.2 / 68.5 / 67.3	79.8	78.5 / 81.1 / 79.8	+ 12.5

Note: P/R/F1 values are reported in the order Precision / Recall / F1-score. DL values represent the macro-averaged F1-score on the independent test set. F1 improvement represents the absolute increase in F1-score from the raw VLM to the VLM-HITL configuration, expressed as percentage points.

The raw zero-shot VLM demonstrated lower classification performance than the DL classifier across all evaluated tasks. Following human-in-the-loop (HITL) refinement, VLM performance improved consistently across all tasks, with F1-score increases of 14.2, 13.8, and 12.5% points for shape/type, color, and surface texture classification, respectively. The VLM-HITL system consequently approached the performance of the DL classifier, particularly for shape/type classification, where the F1-score increased from 72.8% for the raw VLM to 87.0% following HITL refinement, compared with 91.2% for the DL classifier.

### Ablation analysis: contribution of expert-guided HITL refinement

To assess the contribution of the expert-guided human-in-the-loop (HITL) mechanism, the raw zero-shot VLM was compared with the VLM-HITL configuration using the same independent test set of 105 images. This comparison quantified the performance gain associated with adding expert-guided refinement to the zero-shot VLM workflow. As shown in Table 6, HITL refinement consistently improved classification accuracy across all three evaluated tasks. Accuracy increased from 74.0% to 87.0% for shape/type classification, from 71.2% to 83.9% for color classification, and from 67.8% to 79.8% for surface-texture classification, corresponding to absolute gains of 13.0, 12.7, and 12.0% points, respectively.

The analysis also provides a comparative assessment of domain-specific supervised learning relative to zero-shot VLM inference. Compared with the raw VLM, the domain-trained DL classifier achieved accuracy gains of 17.2, 17.3, and 17.2% points for shape/type, color, and surface-texture classification, respectively. Following HITL refinement, the remaining accuracy gap between the VLM-HITL system and the DL classifier was reduced to 4.2, 4.6, and 5.2% points for shape/type, color, and surface texture, respectively.

Overall, the ablation analysis showed a consistent improvement following HITL refinement across all three classification tasks. The accuracy gap between the raw VLM and the DL classifier was reduced from 17.2 to 17.3% points to 4.2–5.2% points after HITL refinement.

**Table 6:** Ablation analysis of the contribution of expert-guided HITL refinement and domain-specific supervised learning.

Task	Experimental comparison	Accuracy (%)	Δ Accuracy (percentage points)
Shape/Type	VLM Raw (zero-shot)	74.0	—
	VLM + HITL	87.0	+ 13.0
	DL classifier	91.2	+ 17.2 vs. VLM Raw
Color	VLM Raw (zero-shot)	71.2	—
	VLM + HITL	83.9	+ 12.7
	DL classifier	88.5	+ 17.3 vs. VLM Raw
Surface Texture	VLM Raw (zero-shot)	67.8	—
	VLM + HITL	79.8	+ 12.0
	DL classifier	85.0	+ 17.2 vs. VLM Raw

Note: Accuracy values were obtained from the same independent test set (*n* = 105). Δ Accuracy represents the absolute difference in accuracy between the indicated configurations and is expressed in percentage points. The VLM Raw → VLM + HITL comparison quantifies the contribution of expert-guided refinement, whereas the VLM Raw → DL comparison describes the performance difference associated with domain-specific supervised learning relative to zero-shot inference.

## Discussion

### Interpretation of quantitative findings

The comparative results provide a clear empirical delineation of the operational strengths and limitations of the two AI paradigms evaluated. The domain-trained EfficientNet-B0 classifier consistently outperformed the zero-shot VLM across all morphological classification tasks, achieving F1-scores between 85% and 91% (Table 3). This outcome supports the hypothesis that task-specific deep learning models outperform generalist AI systems in fine-grained scientific image classification, corroborating a growing body of evidence^1,6,15,21^.

The performance gap is mechanistically attributable to the CNN’s direct optimization on the target domain, enabling it to learn decision boundaries specific to microplastic (MP) optical micrograph features — such as surface texture micropatterns, spectral characteristics of transparent particles, and the aspect-ratio signatures of fibers. This level of quantitative performance is critical for applications requiring high-throughput screening and the generation of large, statistically robust datasets, such as regulatory compliance monitoring or large-scale ecological studies^22^.

Furthermore, the trained CNN’s deterministic output is a critical operational advantage: identical inputs produce identical outputs, ensuring that data generated from time-separated laboratory sessions are strictly comparable — a prerequisite for longitudinal monitoring data quality assurance and scientific reproducibility^23^. This property is absent in VLMs, whose outputs may vary across API calls, API versions, and model updates, representing a substantial reproducibility concern for regulatory-quality datasets.

### Role of HITL guidance and the nature of the VLM-HITL system

The expert-guided HITL mechanism produced consistent and practically significant performance improvements (+ 12 to + 13% points) across all VLM classification tasks (Table 5). It is essential to understand what the HITL mechanism achieves and does not achieve: it is an expert-assisted prompt engineering workflow, not an autonomous learning system. The VLM’s weights do not change; no gradient update occurs; no example is ‘learned.’ Rather, the expert’s perceptual observation is injected as additional textual context (a simple hint), enabling the VLM to apply its general visual reasoning capability more precisely to the specific particle in question. This collaborative approach aligns with emerging trends in other complex scientific fields, where VLMs assist human experts in interpreting data from genomics to remote sensing ^24,25^.

This distinction has important practical implications. First, the HITL benefit depends entirely on the quality and specificity of expert input — it is a structured expert consultation tool, not an AI that improves through use. Second, the resource cost is non-trivial: each HITL intervention requires a trained expert’s attention and a second API call, making it unsuitable for high-throughput screening. Third, the approach is inherently limited by the VLM’s underlying capability — it cannot recover from cases where the VLM lacks sufficient domain knowledge to interpret the hint correctly. Nevertheless, for ambiguous or novel particle types not well-represented in training data, the VLM-HITL system provides a transparent, auditable reasoning trail for each decision, building user trust and aiding scientific documentation^26,27^.

### Comparison with prior architectural approaches

The present results are contextualized relative to the broader deep learning for microplastics landscape. Rermborirak et al.,^13^ achieved > 99% precision using YOLOv8 for fluorescence-stained MP detection — a result achieved under a different imaging modality (Nile Red staining) and narrower classification scope, making direct comparison inappropriate but illustrating the ceiling performance achievable with modality-specific optimization. Royer et al.,^11^ and Yao et al.,^17^ demonstrated segmentation-level multi-class MP identification, which addresses the detection (localization) problem rather than the classification problem addressed here.

Regarding architectural alternatives: Riaz et al.,^15^ demonstrated that lightweight Vision Transformer–MobileNet hybrids with texture-aware attention (TriViT-Lite) achieve state-of-the-art real-time performance on fine-grained classification tasks characterized by texture discrimination — a challenge directly analogous to the rough/smooth discrimination problem in MP texture classification. Similarly, Riaz et al.,^16^ demonstrated multi-scale attention with feature refinement for fine-grained classification under resource constraints. These findings suggest that attention mechanisms sensitive to local texture patterns may yield performance gains over global-average-pooling CNN architectures like EfficientNet-B0 for the texture classification sub-task, where the present model showed the lowest performance (F1 = 85.1%). Future work should evaluate whether texture-aware attention mechanisms improve MP texture classification specifically.

### Practical deployment trade-offs and hybrid workflow

The two systems evaluated here are not mutually exclusive but rather serve complementary roles in a modern environmental laboratory. For environmental monitoring laboratories, the choice between a trained CNN and a VLM-HITL system is not merely architectural but operational:

- The trained CNN is appropriate when: (a) a labelled training dataset is available; (b) high throughput is required (hundreds of particles per day); (c) reproducibility and determinism are critical for data quality assurance; and (d) fixed classification categories are acceptable.
- The VLM-HITL approach is appropriate when: (a) no training data is available; (b) interpretability and natural-language reasoning are required (e.g., for report generation, teaching, or quality-control flagging); (c) small numbers of ambiguous particles require expert-assisted analysis; and (d) flexibility in classification vocabulary is needed.

The hybrid deployment recommended by this study — CNN for bulk screening of hundreds or thousands of particles, VLM-HITL selectively employed to investigate outliers, ambiguous specimens, or novel particle types flagged by the CNN — represents a practically achievable operational workflow that optimizes for both speed and accuracy, leveraging the best of both AI paradigms.

### Accessibility and open science

The deployment of the DL model on Hugging Face Spaces represents a crucial step in democratizing access to advanced analytical technology. By removing barriers related to cost, computational resources, and technical expertise, such open-access platforms empower a wider range of researchers, including those in low-resource settings, to adopt state-of-the-art methods^11^. This aligns with a broader movement towards open science and reproducible research, ensuring that advancements in AI have a tangible and widespread impact on environmental protection efforts^28^.

### Study limitations

While the present study demonstrates the feasibility of AI-assisted microplastic morphological characterization, several limitations should be considered. First, the dataset comprised 700 annotated images, with an independent test set of 105 images and relatively small per-class sample sizes (*n* = 17–28 for shape/type classes), which may limit the precision and generalizability of class-specific performance estimates. Although class weighting was applied during DL training and macro-averaged metrics were used for evaluation, residual effects of class imbalance cannot be completely excluded. Second, the evaluation was conducted using laboratory-derived images acquired under standardized microscopy conditions, and external validation using independent datasets from different laboratories, imaging conditions, geographic locations, and environmental matrices was not performed. Third, the VLM results may be sensitive to prompt formulation because a single predefined prompting framework was evaluated; systematic prompt-robustness testing remains an important direction for future work. Fourth, the VLM-HITL workflow depends on an externally hosted API, introducing practical considerations related to network connectivity, service availability, model-version changes, and potential usage costs, whereas the trained DL classifier can be deployed locally. Finally, the DL and zero-shot VLM approaches have inherently different methodological characteristics: the DL classifier benefits from dataset-specific supervised training, whereas the VLM was evaluated in a zero-shot setting without dataset-specific fine-tuning, with performance subsequently refined through expert-guided HITL feedback. Therefore, direct comparisons should be interpreted in the context of these methodological differences. Future work should focus on larger and more diverse datasets, independent field-based validation, prompt-robustness assessment, locally deployable VLM solutions, and more balanced DL–VLM evaluation frameworks.

## Conclusion

This study presents a comparative evaluation of a domain-trained EfficientNet-B0 classifier and a zero-shot Vision–Language Model (VLM) with expert-guided refinement for morphology-based microplastic characterization from optical microscope images. The results demonstrate that the supervised CNN framework achieved consistently higher performance across shape/type, color, and surface texture classification tasks, while expert-guided refinement substantially improved the performance and interpretability of the zero-shot VLM system. The findings highlight complementary operational advantages between both approaches: the CNN model provides robust and reproducible high-throughput classification, whereas the VLM framework offers flexible, explainable analysis suitable for expert-assisted workflows. Collectively, this work provides practical insight into the trade-offs between trained deep learning pipelines and foundation-model-based approaches for environmental microscopy applications, while establishing a reproducible comparative framework for future research in AI-assisted microplastic analysis.

## Data Availability

The datasets used and/or analyzed during the current study available from the corresponding author on reasonable request.

## Code availability

The training, evaluation, and inference code is publicly available at: [Mahmoudsami/Microplastic_Identification_Tool at main]

The repository includes the full training pipeline (train_model.py), deployed application (app.py), trained EfficientNet-B0 model, class labels, and all required dependencies necessary to reproduce the prediction workflow and evaluate the proposed framework.
